# Calvert Formula Modification for Optimized Carboplatin Dosing in Breast Cancer with Preserved Renal Function

**DOI:** 10.3390/pharmaceutics18040398

**Published:** 2026-03-24

**Authors:** Jihyun Jeon, Jiyeon Jeon, Huong Tra Dang, Eun-hyang Choi, Sang Yull Kang, Hwi-yeol Yun, Jung-woo Chae, Jae Hyun Kim, Soyoung Lee

**Affiliations:** 1 College of Pharmacy, Chungnam National University, 99 Daehak-ro, Yuseong-gu, Daejeon 34134, Republic of Korea; jhjeon952@gmail.com (J.J.); jeonjn@o.cnu.ac.kr (J.J.); huongtra@o.cnu.ac.kr (H.T.D.); jwchae@cnu.ac.kr (J.-w.C.); sy.lee@cnu.ac.kr (S.L.); 2 Department of Pharmacy, Hallym University Dongtan Sacred Heart Hospital, 7 Keunjaebong-gil, Hwaseong 18450, Gyeonggi, Republic of Korea; ehchoi@hallym.or.kr; 3 Department of Surgery, Research Institute of Clinical Medicine, Jeonbuk National University Hospital, Jeonju 54907, Jeonbuk-do, Republic of Korea; sykang@jbnu.ac.kr; 4 Biomedical Research Institute, Jeonbuk National University Hospital, Jeonju 54907, Republic of Korea; 5 Department of Bio-AI Convergence, Chungnam National University, 99 Daehak-ro, Yuseong-gu, Daejeon 34134, Republic of Korea; 6 Senior Health Convergence Research Center, Chungnam National University, Daejeon 34134, Republic of Korea; 7 School of Pharmacy and Institute of New Drug Development, Jeonbuk National University, Jeonju 54907, Jeonbuk-do, Republic of Korea

**Keywords:** Calvert formula, meta-analysis, carboplatin, breast cancer, population pharmacokinetic model

## Abstract

**Background/Objectives**: Although the Calvert formula has been widely used to guide carboplatin dosing, it may yield inaccurate dose predictions in certain patient populations. We aimed to evaluate the adequacy of the conventional Calvert formula and to propose structural modifications to enhance dosing accuracy in breast cancer patients with preserved renal function (CrCL ≥ 55 mL/min). **Methods**: A systematic review and meta-analysis were conducted to integrate published pharmacokinetic models in patients with breast cancer. Two retrospective datasets (*n* = 154) were combined into a single analysis dataset and used to calculate carboplatin doses based on the conventional formulas using creatinine clearance (CrCL) or estimated glomerular filtration rate (eGFR), as well as a modified formula incorporating an additional constant (α). Performance was assessed by the proportion of subjects achieving target area under the curve (AUC) attainment (4–6 mg·min/mL), underexposure (<4 mg·min/mL), and overexposure (≥7 mg·min/mL). All AUC metrics were derived from model-based predictions rather than measured carboplatin concentrations, and no clinical toxicities or efficacy outcomes were used for validation. **Results**: Meta-analysis yielded fixed-effect parameter estimates (*CL*: 131.8 mL/min, *V*_1_: 15.39 L, *K*_12_: 0.002 min^−1^, and *K*_21_: 0.003 min^−1^) with a random effect model. The conventional CrCL-based formula yielded 66.0% target attainment, 22.1% underexposure, and 4.5% overexposure. Switching to eGFR improved attainment to 88.3%, reduced underexposure to 5.8%, and lowered overexposure to 0.65%. A modified formula with α = 1 further decreased underexposure (4.5%) while target attainment and overexposure remained unchanged. **Conclusions**: Replacing CrCL with Chronic Kidney Disease Epidemiology Collaboration (CKD-EPI)-derived eGFR in the Calvert formula markedly improved dosing accuracy, while modest structural modification offered additional benefit. The incremental benefit of α = 1 should be considered hypothesis-generating and requires prospective validation with measured carboplatin concentrations and clinical outcomes before applying it in practice. These findings support adopting eGFR-based dosing in breast cancer and suggest the need for future clinical validation.

## 1. Introduction

Carboplatin has become part of the treatment for specific breast cancer subtypes, notably triple-negative and HER2-positive cancer types [1,2,3], and targets on tumors with defective DNA repair mechanisms. The Calvert formula has been widely used to ensure accurate dosing of carboplatin [4]. This equation was derived using linear regression to characterize the relationship between the measured glomerular filtration rate (GFR) and clearance. In this equation, GFR is originally determined from directly measured renal function using ^51^Cr-EDTA. However, in clinical practice, estimated GFR (eGFR) has been predominantly applied due to the practical challenges associated with direct measurement. One of the most frequently used surrogates is creatinine clearance (CrCL) derived by the Cockcroft–Gault (CG) formula. However, CrCL may differ substantially from measured GFR, and applying the Calvert formula with this can result in inaccurate dose predictions [5,6,7,8]. Numerous studies have proposed refinements of eGFR to improve carboplatin dose prediction. In particular, the use of the Chronic Kidney Disease Epidemiology Collaboration (CKD-EPI) equation has been reported in several studies as a more reliable approach [5,6,7].

A substantial proportion of breast cancer patients present with preserved or near-normal renal function. Launay-Vacher et al. reported that approximately 38.6% of patients exhibit normal renal function and more than 43% have moderate renal function, indicating that the majority of patients fall within ranges where a small bias in renal function estimation may translate into clinically relevant dosing differences [9]. This highlights the importance of optimizing renal-function-based dosing strategies in this population.

Beyond refining eGFR, reassessment of the Calvert formula itself could be especially warranted in breast cancer, where carboplatin has been used off-label [10]. In contemporary clinical practice, carboplatin is commonly incorporated into treatment regimens for specific breast cancer subtypes, particularly triple-negative and HER2-positive breast cancer [1,2,3]. However, carboplatin dosing recommendations in breast cancer rely on the conventional Calvert formula that was extrapolated from other tumor types, and there is no specific equation modified or established for breast cancer dosing. Recent pharmacokinetic studies could provide an opportunity to evaluate the predictive accuracy of the Calvert formula in this population.

Model integration through a meta-analysis of published population pharmacokinetic (PopPK) models could offer a novel framework for addressing this need. This approach enables a more robust characterization of carboplatin pharmacokinetics by synthesizing parameter estimates across diverse studies and provides an evidence-based foundation for reevaluating dose calculation strategies.

We evaluated the adequacy of the conventional Calvert formula when applied to eGFR derived by CKD-EPI and, at the same time, reassessed the formula itself to explore potential modifications for more accurate dose prediction. To achieve this, we integrated published PopPK models, including models developed in mixed tumor populations, through meta-analysis and applied the integrated model to retrospectively collected breast cancer datasets for simulation-based area under the curve (AUC) prediction. Visualization of the study process can be seen in Supplementary Materials (Figure S1). These analyses were based on model predicted exposures and were not validated against measured carboplatin concentrations, clinical efficacy or toxicity outcomes.

## 2. Materials and Methods

### 2.1. Systematic Review and Meta-Analysis for Model Integration

A systematic literature review was conducted to collect PopPK models of carboplatin in patients with breast cancer. The PubMed database of the U.S. National Library of Medicine was searched using the keywords carboplatin(tw) AND pharmacokinetics(tw) AND (NONMEM[tw] OR population[tw]). Screening was first performed based on titles and abstracts. The inclusion criteria were as follows: (a) studies including patients with breast cancer, (b) availability of PopPK model parameters, and (c) adult study populations.

From all identified studies, we extracted information on typical values of pharmacokinetic parameters in the PopPK model. Only fixed-effect (typical) parameter estimates from the final published models were integrated in the meta-analysis. Covariate effects were not incorporated, as individual-level data were not available and no covariates were retained in the final structural models of the included studies. Therefore, the integrated model represents a typical-patients framework rather than a full covariate-adjusted population model.

Meta-analysis of the fixed effect parameters was performed using a random effects model assumption, which considered both within-study error in estimating the effect in each study and the heterogeneity of the studies. This analysis was performed using Meta library [11] in R v.4.3.2 and RStudio v.2023.09.1+494.

### 2.2. Data Collection

Two independent data sources were used in this study. Dataset 1 was extracted from the Electronic Health Record (EHR) database of Jeonbuk National University Hospital, Republic of Korea. These data were collected under retrospective study approved by the Institutional Review Board (IRB No. 2024-03-026-001). This dataset included adult patients who received carboplatin between October 2019 and April 2024.

Dataset 2 consisted of publicly available data derived from a retrospective study exempted from informed consent requirements in accordance with Portuguese Law No. 21/2014. The study protocol was approved by the Ethics Committee of ULSAAve (Ref. 90/2024). This dataset included all carboplatin administrations performed in the outpatient oncology department between 1 January and 31 December 2023, and was extracted from the hospital electronic system in July 2024 [12].

Both datasets contained CrCL calculated using the CG equation, eGFR derived from the CKD-EPI equation, body surface area (BSA), and the target AUC for the first cycle of carboplatin therapy. These two datasets were merged into a single integrated dataset and used for dose calculation based on both the conventional and modified Calvert equations.

To ensure consistency with the renal function range represented in the integrated PopPK models and to avoid extrapolation, patients with CrCL < 55 mL/min were excluded from the analysis. In the context of this study, the term “preserved renal function” refers to CrCL ≥ 55 mL/min, reflecting the renal function distribution of the source studies included in the meta-analysis. Therefore, this study focused on patients within this renal function range.

### 2.3. Evaluation of Modified Calvert Formula

Carboplatin doses were calculated using three approaches: the conventional formula with CrCL (1), the conventional formula with BSA-adjusted eGFR (2), and a modified formula incorporating an additional constant α (3).

Dose (mg) = Target AUC∙(CrCL + 25)(1)

Dose (mg) = Target AUC∙(BSA readjusted eGFR + 25)(2)

Dose (mg) = Target AUC∙(BSA readjusted eGFR + 25 mL/min +α [mL/min])(3)

where target AUC denotes the target area under the plasma concentration–time curve (mg·min/mL), and BSA readjusted eGFR (mL/min) was derived as eGFR (mL/min/1.73 m^2^) × BSA (m^2^)/1.73.

The parameter *α* was introduced as an exploratory sensitivity factor to account for potential systematic discrepancies between estimated renal function (eGFR or CrCL) and true carboplatin clearance. The lower bound represents an extreme scenario in which clearance is predominantly renal, minimizing non-renal contribution. The upper bound represents a plausible systematic overestimation or underestimation of true clearance. In clinical practice, ±25 mL/min is comparable to known variability and bias between estimated and measured renal function. Thus, α serves as a sensitivity adjustment rather than a mechanistically derived physiological constant. The α values were varied from −25 to +25 mL/min in increments of 1 mL/min.

All derived doses from (1–3) were applied to the PopPK model (integrated through meta-analysis) to predict exposure. The AUC over 24 h was estimated under the assumption of a single 3 h infusion. Simulations were based on typical values of parameters only; inter–individual variability (IIV) in clearance, central volume of distribution and other pharmacokinetic (PK) parameters was not incorporated. Variability in typical parameter estimates was assessed using confidence intervals derived from the meta-analysis. The target AUC was set at 4–6 mg·min/mL, as this range is most appropriate in previously treated patients, while escalation beyond 7 mg·min/mL increases myelotoxicity without improving response [13]. The optimal value of α was determined by evaluating three AUC-based criteria: (1) the proportion within the target range (4–6 mg·min/mL), (2) the proportion below the target (<4 mg·min/mL, indicating reduced efficacy), and (3) the proportion above the target (≥7 mg·min/mL, indicating increased toxicity). Results from the modified formula (Equation (3)) were compared against those from the conventional formula with eGFR (Equation (2)) to identify the α value that maximizes efficacy while minimizing toxicity. The simulation was performed using NONMEM v.7.5.0, and the statistical summary or visualization was performed using R v.4.3.2.

## 3. Results

### 3.1. Systematic Review and Meta-Analysis for Model Integration

A total of 137 records were initially identified. Screening was first performed based on titles and abstracts. After exclusion of non-English publications and animal studies, 77 articles remained for full-text review. Among them, 26 studies were excluded due to inconsistency with the study objectives, and 12 studies were excluded due to lack of human PK data. The remaining 39 articles underwent further evaluation. Studies that did not include breast cancer patients, lacked adequate pharmacokinetic parameter information, did not clearly describe model structure and analytical methods, or did not use a population-based modeling approach were excluded. Ultimately, three studies were included in the meta-analysis (Table S1). A flow chart of the systematic review can be seen in the Supplementary Material (Figure S2).

The populations used for model development in the included studies were those with breast, non-small cell lung, ovarian, and germ cell cancers. When integrating renal function as CrCL values reported across these studies, the overall range was 55–451 mL/min, with the lower bound of 55 mL/min applied as a cutoff for the data analysis in this study.

All collected models were described using a two-compartment structure (Figure S3). Fixed-effect parameter estimates, together with their relative standard errors (RSEs) or standard errors (SEs), as well as the number of subjects (N) included in each study, were extracted for meta-analysis.

The reported parameters included clearance (*CL*), central volume of distribution (*V*_1_), peripheral volume of distribution (*V*_2_), intercompartmental clearance (*Q*), and/or first-order intercompartmental rate constants (*K*_12_ *= Q/V*_1_ and *K*_21_ *= Q/V*_2_).

Statistically significant heterogeneity was observed for *CL*, *V*_1_, *K*_12_, and *K*_21_ (all *p* < 0.0001) in the meta-analysis. Heterogeneity was quantified using I^2^ statistics (*CL*: 95.4%, *V*_1_: 99.8%, *K*_12_: 99.8%, *K*_21_: 99.8%); therefore, a random-effects model was applied to account for between-study variability. To enable application of the Calvert formula, all parameter units were standardized, resulting in the following typical values: *CL*, 131.8 mL/min (= 7.91 L/h); *V*_1_, 15.39 L; *K*_12_, 0.002 min^−1^ (=0.12 h^−1^); and *K*_21_, 0.003 min^−1^ (=0.18 h^−1^) (Table 1 and Figure S4).

### 3.2. Analysis Dataset

Datasets 1 and 2 are summarized in Table S2. Out of a total of 167 subjects that were collected from data sources, 13 subjects were excluded from dataset 2 after applying the lower bound from the meta-analysis (CrCL < 55 mL/min). In total, 154 subjects were included in this analysis. For dataset 1, two subjects with an eGFR of 104.36 ± 17.03 mL/min/1.73 m^2^ (range 92.31, 116.40 mL/min/1.73 m^2^) were included for the AUC target of five. For the AUC target of six, 58 subjects were available, with a mean (±SD) eGFR of 102 ± 9.11 mL/min/1.73 m^2^ (range, 79.38–121.27 mL/min/1.73 m^2^). In dataset 2, 57 subjects were included for the AUC target of 5 mg·min/mL, with a mean (±SD) eGFR of 98.3 ± 13.3 mL/min/1.73 m^2^ (range, 61–120 mL/min/1.73 m^2^). For the AUC target of 6 mg·min/mL, 37 subjects were available, with a mean (±SD) eGFR of 97.6 ± 14.2 mL/min/1.73 m^2^ (range, 66–119 mL/min/1.73 m^2^).

### 3.3. Evaluation of Conventional and Modified Calvert Formula

**Figure 1:**
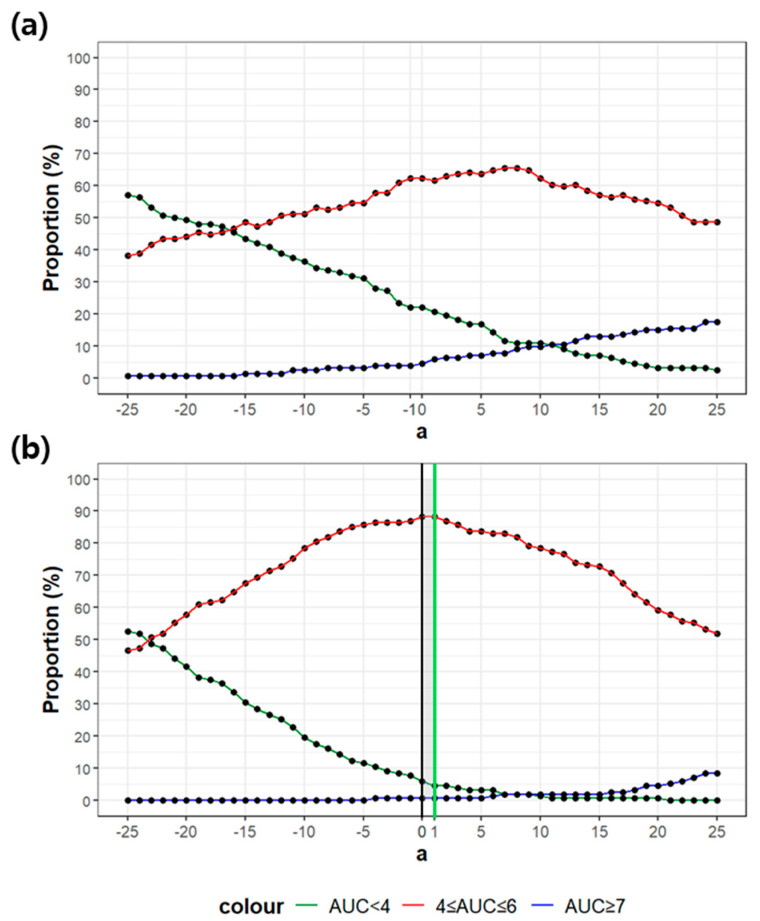
Target attainment (%) plot when applying Calvert formula with (**a**) Creatinine Clearance and (**b**) estimated glomerular filtration rate. α represents the constant added to the conventional Calvert formula; points and lines depict the proportion of patients achieving target attainment (%) across α values ranging from −25 to 25. Colors indicate predefined criteria: red represents the proportion of patients attaining the target AUC range of 4–6 mg·min/mL, green denotes the proportion with AUC < 4 mg·min/mL, and blue indicates the proportion with AUC ≥ 7 mg·min/mL; grey shaded area and neon-green line represent optimal α.

The conventional formula with CrCL (1) achieved a target AUC attainment of 66.0%, with 22.1% of patients underexposed (AUC < 4 mg·min/mL) and 4.5% overexposed (AUC ≥ 7 mg·min/mL). In contrast, the conventional formula with eGFR (2) markedly improved performance, yielding 88.3% target attainment, while reducing subtherapeutic exposure to 5.8% and supratherapeutic exposure to 0.65% (Figure 1a).

These results were used to establish criteria for setting optimal α in (3): subtherapeutic exposure < 5.8%, target attainment > 88.3%, and supratherapeutic exposure < 0.65%. Within this framework, the modified formula with α = 1 satisfied these criteria. Notably, target attainment and supratherapeutic exposure were identical to the eGFR-based formula, whereas subtherapeutic exposure was further reduced to 4.5% (Table S4 and Figure 1b). Accordingly, the theoretically optimized equation can be expressed as:

Dose (mg) = Target AUC∙(BSA readjusted eGFR + 26)(4)

## 4. Discussion

The findings of this study suggest that the conventional Calvert formula may result in inaccurate carboplatin dosing in patients with breast cancer with relatively preserved renal function. Given that a large portion of breast cancer patients present with normal or near-normal renal function, even a small bias in renal function estimation may translate into clinically meaningful dosing differences. In such populations, reliance on creatinine clearance-based estimation may increase the risk of inappropriate dosing and suboptimal exposure. Analysis showed that 22.1% of patients were predicted to fall below the target AUC range when using CrCL, whereas this proportion decreased to 5.8% when CKD-EPI eGFR was used, corresponding to an absolute reduction in underexposure of 16.3%.

In the present study, replacing CrCL with eGFR derived from the CKD-EPI equation in the Calvert formula substantially improved dosing prediction. The improvement was demonstrated by higher target attainment (88.3% vs. 66%) and a lower risk of both underexposure and overexposure. Notably, compared with eGFR-based dosing, CrCL-based dosing resulted in a 1.3-fold lower target attainment and an approximately eight-fold higher rate of supratherapeutic exposure (4.5% vs. 0.65%). These findings reinforce the validity of adopting eGFR, particularly CKD-EPI-derived values, as the preferred renal function marker when applying the Calvert formula in clinical practice.

The integration of the Korean and Portuguese clinical datasets was considered appropriate for this analysis. Because carboplatin exposure was recalculated using renal function metrics and target AUC values rather than observed dosing practices, the pharmacokinetic simulations were primarily driven by renal function and target exposure rather than institutional treatment strategies. Furthermore, carboplatin pharmacokinetics are predominantly determined by renal clearance and have not been reported to demonstrate meaningful race-related differences, supporting the pooling of datasets from different geographic regions. Regarding creatinine assay standardization, both centers reported CKD-EPI derived eGFR, which presupposes the use of IDMS-traceable creatinine assays. Although residual inter-laboratory variability may exist due to differences in analytical platforms, such differences are generally small following global creatinine standardization and were unlikely to materially affect the conclusions.

Although our additional adjustment incorporating the constant α offered only a modest improvement, the approach still reduced subtherapeutic exposure without compromising safety. The primary improvement observed in this study was achieved by replacing CrCL with CKD-EPI–derived eGFR in the Calvert formula. The α = 1 refinement produced a small reduction in underexposure while maintaining similar overall target attainment. Therefore, this adjustment should be interpreted as a potential optimization rather than a clinically validated modification of the dosing strategy. Further validation using larger, independent, heterogeneous cohorts and correlation with clinical outcomes will be required before clinical implementation.

The strengths of this study lie in its methodological framework. Our analysis incorporated an updated estimator for renal function and reassessed the Calvert formula itself using contemporary clinical datasets. In addition, the use of meta-analysis to integrate population pharmacokinetic parameters allowed the derivation of robust typical values, reducing the uncertainty associated with individual studies. The inclusion of both electronic health record-derived data and external retrospective datasets further enhanced the external validity of the analysis.

Nevertheless, several limitations should be acknowledged. First, the evaluation was primarily based on pharmacokinetic simulations rather than clinical outcome data; therefore, the relationship between predicted AUC and actual treatment outcomes, including efficacy and toxicity, remains to be prospectively validated. Second, the AUC targets used in this study were primarily derived from exposure–response relationships established in ovarian cancer populations, while breast cancer-specific exposure–response data remain limited. Therefore, extrapolation of these AUC targets to breast cancer represents an assumption and should be interpreted cautiously. Third, only patients with preserved renal function (CrCL ≥ 55 mL/min) were included in this analysis, and the findings may not be directly generalizable to patients with moderate or severe renal impairment. Additionally, although the CKD-EPI equation is considered an improved estimator of renal function compared with conventional CrCL, creatinine-based estimators may still be influenced by factors such as muscle mass and body composition. This may lead to potential misestimation of renal function, particularly in oncologic populations where sarcopenia is common.

In addition, the PopPK parameters used in the integrated model were derived from studies that included mixed tumor populations rather than exclusively breast cancer patients. Although this may raise concerns regarding the representativeness of pharmacokinetic parameters for breast cancer populations, tumor type was not identified as a significant covariate influencing carboplatin clearance or volume of distribution in the original PopPK models, suggesting that pharmacokinetic differences across tumor types are likely minimal.

Furthermore, because the integrated model was constructed using only typical parameter estimates, potential covariate-related variability could not be evaluated. Although tumor type was not retained as a significant covariate in the original models, subtle tumor-specific pharmacokinetic differences cannot be completely excluded. In addition, detailed information regarding breast cancer molecular subtypes was not consistently available in the retrospective datasets, and therefore could not be incorporated into the analysis. Also, potential differences between the Korean and Portuguese cohorts, such as ethnicity, body size, creatinine assay methods, and clinical practice patterns, were not explicitly modeled, which may have introduced additional variability. Moreover, given the small number of studies included in the meta-analysis (*n* = 3), estimates of between-study heterogeneity may be unstable and I^2^ values may appear inflated.

In conclusion, this study demonstrates that substituting CrCL with CKD-EPI-derived eGFR in the Calvert formula markedly improves dose prediction in breast cancer patients with preserved renal function. A theoretical structural modification of the formula provides additional incremental benefit, although further validation will be required before clinical implementation. Future research should focus on evaluating the relationship between predicted exposure and clinical outcomes across broader patient populations with varying renal function and tumor types.

## Figures and Tables

**Table 1:** Population pharmacokinetic parameter estimates from meta-analysis.

	Reference 1 [14]		Reference 2 [15]		Reference 3 [16]		Meta-Analysis
	Estimate	RSE (%CV)	Estimate	SE	Estimate	RSE (%CV)	Estimate [95% CI]
***CL* (L/h)**	8.33	19.1	6.99	0.74	8.38	1.41	7.91 [7.07–8.84]
***V*** **_1_ (L)**	16.3	17	14.4	0.99	15.4	1.79	15.39 [14.37–16.48]
***V*** **_2_ (L)**			24.8	0.46			
***Q* (L/h)**			1.22	2.7			
***K*** **_12_ (/h)**	0.104	37.8			0.135	7.85	0.12 [0.09–0.15]
***K*** **_21_** **(/h)**	0.171	49.2			0.215	5.91	0.18 [0.14–0.24]

RSE, relative standard error; SE, standard error; *CL*, clearance; *V*_1_, central volume of distribution; *V*_2_, peripheral volume of distribution; *Q*, intercompartmental clearance; *K*_12_ and *K*_21_, elimination rate constants.

## Data Availability

The data presented in this study are available on request from the corresponding author. (The data are not publicly available due to privacy or ethical restrictions).

## Supplementary Materials

The following supporting information can be downloaded at https://www.mdpi.com/article/10.3390/pharmaceutics18040398/s1: Figure S1. Graphical abstract of research process, Figure S2. Flow chart of systematic review, Figure S3. Population PK model scheme, Figure S4. Result of meta-analysis of population PK models, Table S1. Summary of key characteristics of population pharmacokinetic studies included in the meta-analysis, Table S2. Raw dataset status, Table S3. Analysis dataset status, Table S4. Target attainment proportion across α values ranging from −25 to 25.
