# Supplementary material for: Calvert Formula Modification for Optimized Carboplatin Dosing in Breast Cancer with Preserved Renal Function
Source: Pharmaceutics. 2026 Mar 24;18(4):398. doi: 10.3390/pharmaceutics18040398 (PMC13118422; doi:10.3390/pharmaceutics18040398)
Supplement: Supplementary file 1 [file pharmaceutics-18-00398-s001.zip › pharmaceutics-4136843-supplementary.pdf]

Supplementary

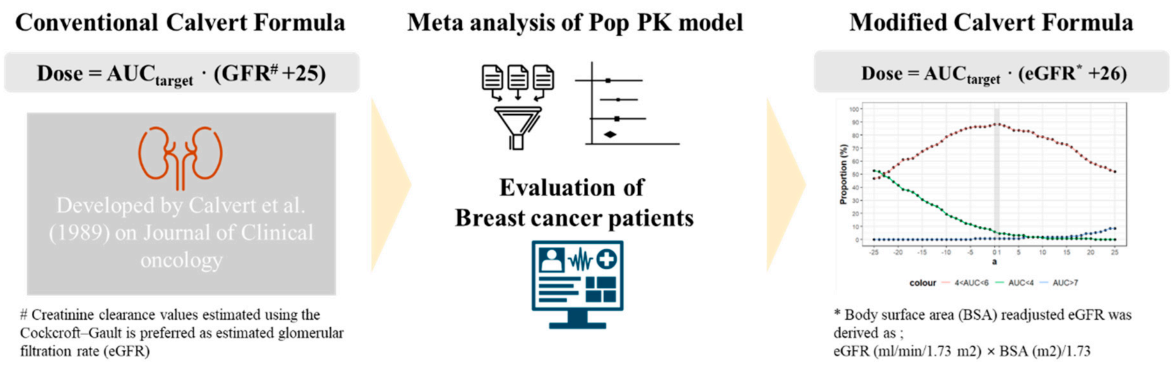

Figure S1 Graphical abstract of research process

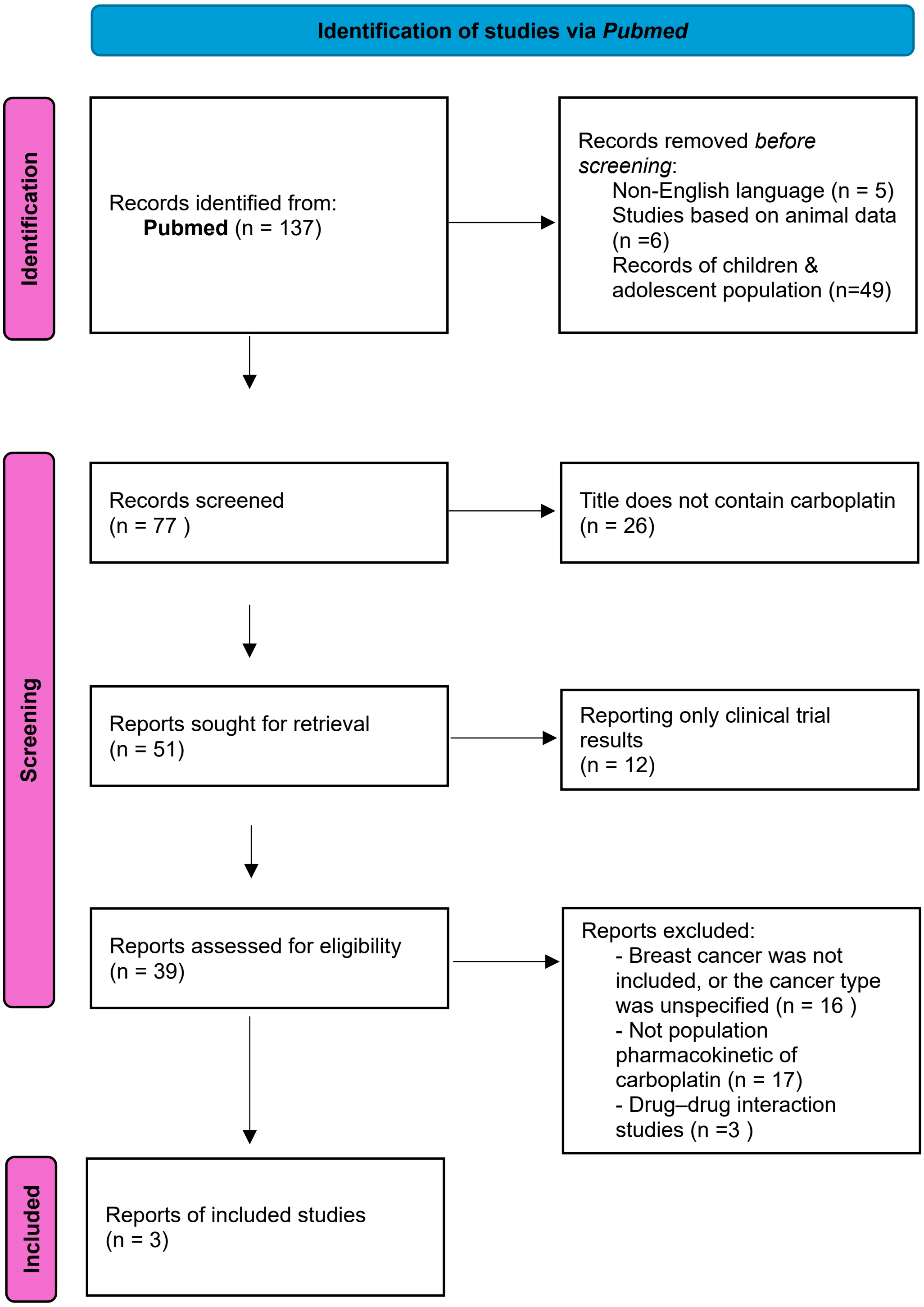

Figure S2 Flow chart of Systematic review

This flow chart is following statement of Preferred Reporting Items for Systematic reviews and Meta-Analyses (PRISMA) 2020

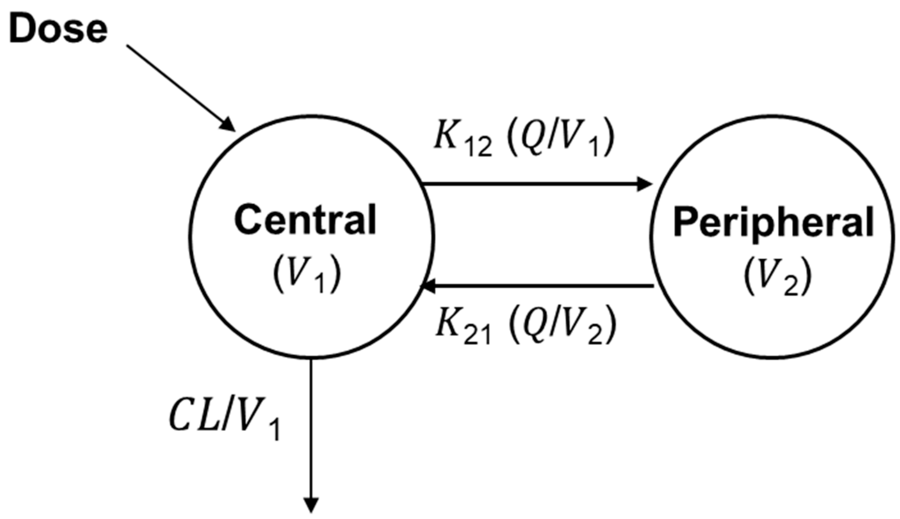

Figure S3 Population PK model scheme

CL, Clearance ; V1, central volume of distribution; V2, Peripheral volume of distribution; Q, intercompartmental clearance; K12 and K21, Elimination rate constant

1 . Meta analysis of CL

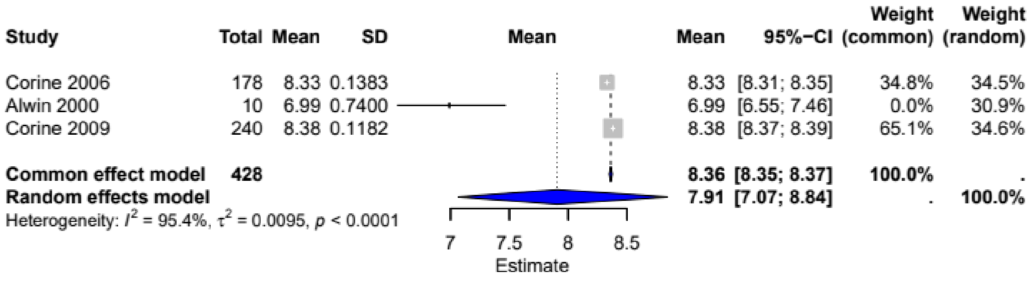

2 . Meta analysis of V1

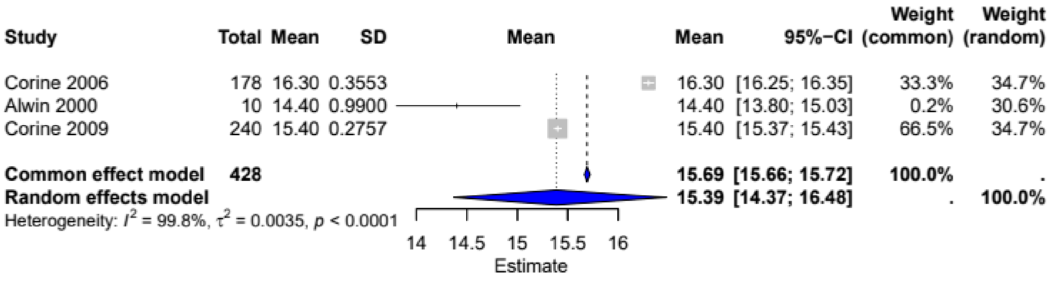

3 . Meta analysis of K12

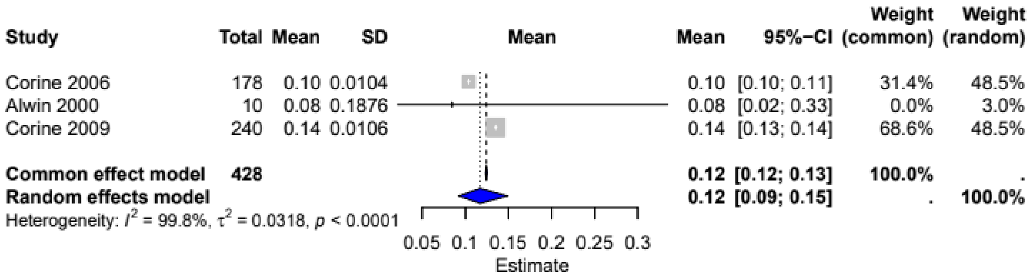

4 . Meta analysis of K21

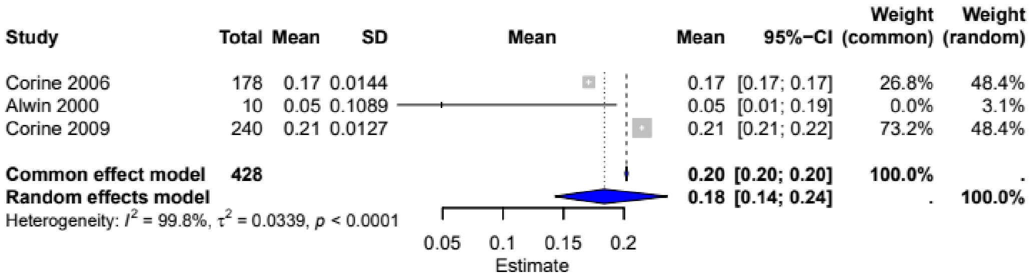

Figure S4 Result of Meta analysis of population PK models

CL, Clearance ; V1, central volume of distribution; V2, Peripheral volume of distribution; Q, intercompartmental clearance; K12 and K21, Elimination rate constant

Table S1 Summary of key characteristics of the population pharmacokinetic studies included in the meta-analysis

	**Reference 1 [13]**	**Reference 2 [14]**	**Reference 3 [15]**
**N of subjects**	178	10	240
**Cancer types**	Non-small cell lung cancer, Ovarian cancer, Breast cancer, Germ cell cancer, Metastatic ovarian cancer	Breast cancer Ovarian cancer Germ cell cancer	Non-small cell lung cancer, Ovarian cancer, Breast cancer Metastatic breast cancer, Refractory germ cell cancer, Metastatic ovarian cancer, Epithelial breast cancer
**CrCL range (calculated with the Cockcroft-Gault formula (mL/min))**	55-451	63.6 - 143	55–451
**PopPK structural model**	2-compartment with first order elimination without covariate	2-compartment with first order elimination without covariate	2-compartment with first order elimination without covariate
**Treatment regimen**	Combination (CTC, Cyclophosphamide, thiotepa, and carboplatin)	Combination (CTC, Cyclophosphamide, thiotepa, and carboplatin)	Combination (CTC, Cyclophosphamide, thiotepa, and carboplatin)

Table S2 Raw dataset status

	Dataset 1<br>Mean ± SD (Range)	Dataset 2<br>Mean ± SD (Range)
Number of subjects	60	107
Target AUC	(5.00–6.00)	(5.00–6.00)
CrCL (Cockcroft-Gault)	97.32 ± 21.61 (57.53–171.00)	84.03 ± 28.19 (26.00–184.00)
BMI	23.77 ± 3.43 (16.31–33.07)	24.63 ± 4.67 (14.40–38.20)
Body weight	58.43 ± 8.72 (42.80–75.30)	57.62 ± 5.21 (45.00–70.00)
eGFR (CKD-EPI)	102.08 ± 9.23 (79.38–121.27)	93.72 ± 18.30 (25.00–120.00)
BSA	1.55 ± 0.12 (1.27–1.80)	1.68 ± 0.18 (1.29–2.13)

AUC, area under curve; CrCL, creatinine clearance based on Cockcroft-Gault equation; BMI, body max index; eGFR, estimated glomulular filtration rate; CKD-EPI, Chronic Kidney Disease Epidemiology Collaboration; BSA, body surface area

Table S3 Analysis dataset status

Case	# of subject	Target AUC	Mean of eGFR	SD of eGFR	Range of eGFR (min-max)
Dataset 1	2	5	104.36	17.03	(92.31, 116.40)
Dataset 1	58	6	102.00	9.11	(79.38 – 121.27)
Dataset 2	57	5	98.30	13.26	(61 – 120)
Dataset 2	37	6	97.57	14.17	(66 – 119)

AUC, Area under the curve (mg·min/mL); eGFR, estimated glomerular filtration rate(ml/min/1.73 m^2) ; SD, standard deviation

Table S4 Target attainment proportion across α values ranging from −25 to 25

α	N	Proportion AUC < 4	4 ≤ AUC ≤ 6	AUC≥ 7
-25	154	0.525974026	0.4675325	0
-24	154	0.519480519	0.474026	0
-23	154	0.487012987	0.5064935	0
-22	154	0.474025974	0.5194805	0
-21	154	0.441558442	0.5519481	0
-20	154	0.415584416	0.5779221	0
-19	154	0.383116883	0.6103896	0
-18	154	0.376623377	0.6168831	0
-17	154	0.363636364	0.6233766	0
-16	154	0.337662338	0.6493506	0
-15	154	0.305194805	0.6753247	0
-14	154	0.285714286	0.6948052	0
-13	154	0.266233766	0.7142857	0
-12	154	0.253246753	0.7272727	0
-11	154	0.227272727	0.7532468	0
-10	154	0.194805195	0.7857143	0
-9	154	0.175324675	0.8051948	0
-8	154	0.162337662	0.8181818	0
-7	154	0.142857143	0.8376623	0
-6	154	0.123376623	0.8506494	0
-5	154	0.116883117	0.8571429	0
-4	154	0.103896104	0.8636364	0.006493506
-3	154	0.090909091	0.8636364	0.006493506
-2	154	0.084415584	0.8636364	0.006493506
-1	154	0.077922078	0.8701299	0.006493506
0	154	0.058441558	0.8831169	0.006493506
1	154	0.045454545	0.8831169	0.006493506
2	154	0.045454545	0.8701299	0.006493506
3	154	0.038961039	0.8571429	0.006493506
4	154	0.032467532	0.8376623	0.006493506
5	154	0.032467532	0.8376623	0.006493506
6	154	0.032467532	0.8311688	0.012987013
7	154	0.019480519	0.8311688	0.019480519
8	154	0.019480519	0.8181818	0.019480519
9	154	0.019480519	0.7922078	0.019480519
10	154	0.012987013	0.7857143	0.019480519
11	154	0.006493506	0.7727273	0.019480519
12	154	0.006493506	0.7662338	0.019480519
13	154	0.006493506	0.7402597	0.019480519
14	154	0.006493506	0.7337662	0.019480519
15	154	0.006493506	0.7272727	0.019480519
16	154	0.006493506	0.7077922	0.025974026
17	154	0.006493506	0.6753247	0.025974026
18	154	0.006493506	0.6428571	0.032467532
19	154	0.006493506	0.6168831	0.045454545
20	154	0.006493506	0.5909091	0.045454545
21	154	0	0.5779221	0.051948052
22	154	0	0.5584416	0.058441558
23	154	0	0.5519481	0.071428571
24	154	0	0.5324675	0.084415584
25	154	0	0.5194805	0.084415584

AUC, Area under the curve (mg·min/mL); α, constant added to the conventional Calvert formula; N, Number of subjects
